# Facial Cartilaginous Reconstruction—A Historical Perspective, State-of-the-Art, and Future Directions

**DOI:** 10.3389/fsurg.2021.680186

**Published:** 2021-08-16

**Authors:** Zita M. Jessop, Adam Hague, Thomas D. Dobbs, Kenneth J. Stewart, Iain S. Whitaker

**Affiliations:** ^1^Reconstructive Surgery and Regenerative Medicine Research Group, Swansea University Medical School, Swansea, United Kingdom; ^2^The Welsh Centre for Burns and Plastic Surgery, Morriston Hospital, Swansea, United Kingdom; ^3^Department of Plastic and Reconstructive Surgery, Royal Hospital for Sick Children, Edinburgh, United Kingdom

**Keywords:** facial reconstruction, cartilage, tissue engineering, regenerative medicine, plastic and reconstructive surgery

## Abstract

**Importance:** Reconstruction of facial deformity poses a significant surgical challenge due to the psychological, functional, and aesthetic importance of this anatomical area. There is a need to provide not only an excellent colour and contour match for skin defects, but also a durable cartilaginous structural replacement for nasal or auricular defects. The purpose of this review is to describe the history of, and state-of-the-art techniques within, facial cartilaginous surgery, whilst highlighting recent advances and future directions for this continually advancing specialty.

**Observations:** Limitations of synthetic implants for nasal and auricular reconstruction, such as silicone and porous polyethylene, have meant that autologous cartilage tissue for such cases remains the current gold standard. Similarly, tissue engineering approaches using unrelated cells and synthetic scaffolds have shown limited *in vivo* success. There is increasing recognition that both the intrinsic and extrinsic microenvironment are important for tissue engineering and synthetic scaffolds fail to provide the necessary cues for cartilage matrix secretion.

**Conclusions and Relevance:** We discuss the first-in-man studies in the context of biomimetic and developmental approaches to engineering durable cartilage for clinical translation. Implementation of engineered autologous tissue into clinical practise could eliminate donor site morbidity and represent the next phase of the facial reconstruction evolution.

## Introduction

The reconstruction of facial defects continues to pose a significant surgical challenge. Facial disfigurements, including nasal and auricular defects following trauma, burns, skin cancer resection, and congenital conditions requiring reconstruction affect 569,000 (or 1 in 111) people in the United Kingdom (1). Facial deformity and scarring can have a devastating effect on an individual's appearance, psychological health and subsequently on their quality of life (2–4). When reconstructing facial defects all rungs of the reconstructive ladder must therefore be utilised to replace tissue like-for-like and ultimately optimise both the functional and cosmetic outcomes.

Skin grafts and local flaps are widely used within facial plastic surgery where there is a cutaneous defect (5–8). For larger areas, or in sites of reduced vascularity, the only viable option may be reconstruction with a free flap (9, 10). The anterolateral thigh and radial forearm flaps have traditionally been used in head and neck reconstruction with the free fibula flap also providing an autologous source of bone (11). Revision procedures in order to de-bulk the flap and improve cosmesis, however, are not uncommon (10, 12).

Despite advances in surgical techniques, one area that remains a challenge within facial plastic surgery is the reconstruction of defects where there is a deficiency of cartilage. This is in part due to cartilage, unlike other tissue types, having no ability for repair and regeneration. Although synthetic options for nasal (13) and auricular (14) reconstruction have been described, the use of autologous tissue for such cases remains the current gold standard (Table 1) (15). Such grafts can be harvested from the ear, septum or costal cartilages. However, as with all autologous reconstruction there is a limited amount of tissue available as well as the potential for donor site morbidity. In order to address this problem recent advances in tissue engineering and 3D bioprinting aim to provide clinicians with the ability to create autologous cartilage within the laboratory setting, potentially changing the field of facial reconstructive surgery forever (16, 17). The purpose of this review is to describe the history of, and current techniques within, facial cartilaginous surgery whilst also outlining future directions for this continually advancing specialty.

**Table 1:** Uses of cartilage grafting in facial reconstructive surgery.

**Location**	**Condition examples**	**Indication**
Nose • Ala • Side wall •Tip	Skin cancer, trauma, congenital, aesthetic	Relative
Ear	Skin cancer, trauma, congenital	Absolute
Eyelid (tarsal plate)	Skin cancer, trauma	Absolute
Orbital wall	Trauma	Relative

## The Birth of Facial Plastic Surgery

**Figure 1:**
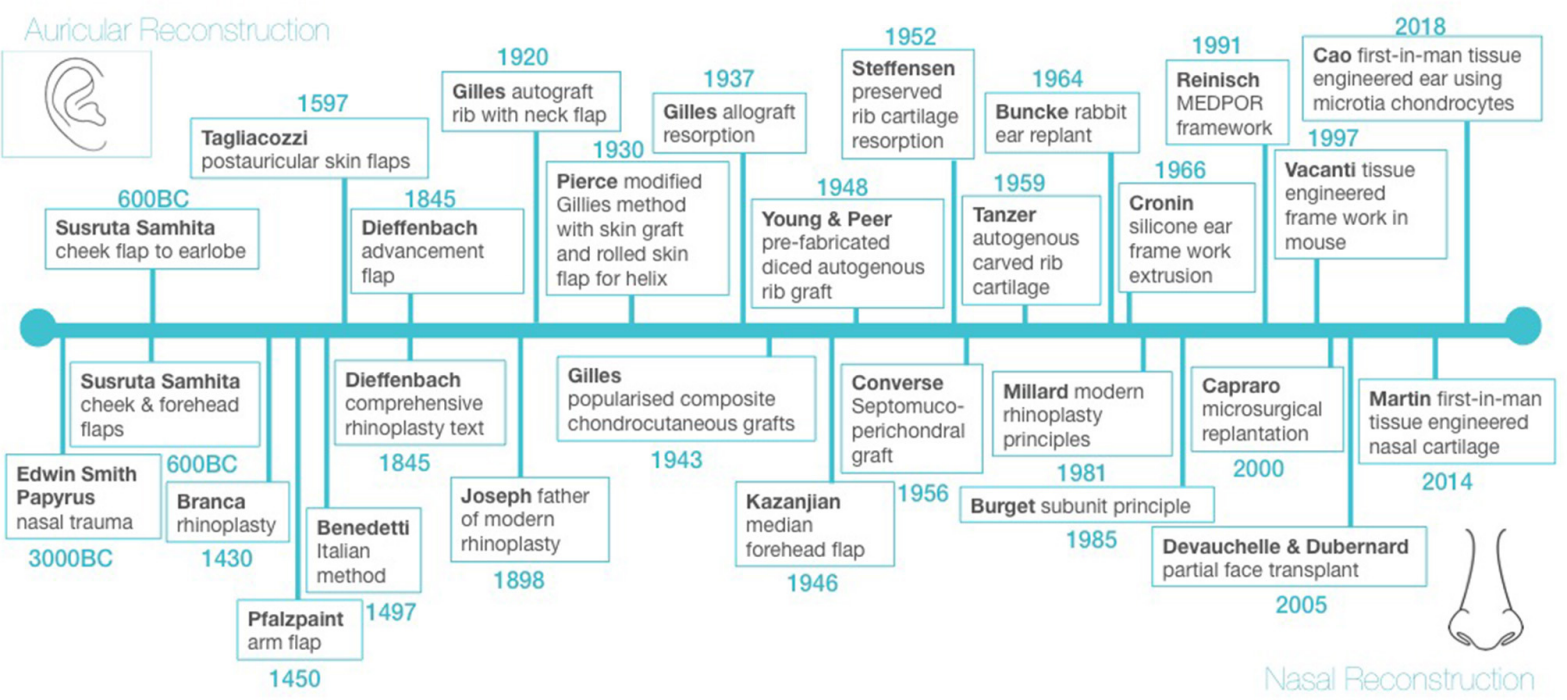
History of cartilaginous based facial reconstruction.

The first mention of the treatment of facial trauma is found in the Edwin Smith Surgical Papyrus dated circa 3000 BC (18). Total nasal reconstruction and partial auricular reconstruction around 600 BC constitute the first chapter in the history of not only facial reconstruction but plastic reconstructive surgery in general (Figure 1) (19, 20). These principles were handed down through civilisations, with the renaissance of the 14th century bringing a rebirth of reconstructive surgery, particularly through the work of Italian surgeons Branca (1430) and Benedetti (1497) who were instrumental in developing rhinoplasty techniques and Tagliacozzi (1597) who developed numerous auricular reconstructive techniques (Figure 1).

World War I produced the greatest number of facial injuries and burns in the history of warfare and led to the development of modern facial reconstruction by Gillies (20). Gillies developed tubed pedicled flaps for facial reconstruction, as well as pioneering rib autografts and allografts for auricular reconstruction and composite chondrocutaneous grafts for nasal reconstruction (Figure 1) (21–24). These early cartilage grafts did, however, suffer from significant rates of resorption (22). More recent attempts to use irradiated allogenic grafts from cadaveric donors also demonstrated over 70% resorption, as well as concerns regarding risk of disease transmission (23, 24).

## Alternatives to Autografts in Facial Cartilaginous Reconstruction

The use of alloplastic implants for facial reconstruction has been widely described. These synthetic materials have pores of varying sizes that, when present, allow for tissue ingrowth but can also result in bacterial colonisation and subsequent infection (13). Cronin (14) was the first to introduce silicone ear frameworks. Although silicone is easily moulded into the desired shape, its nonporous structure prevents tissue ingrowth which can result in capsule formation and subsequent implant distortion (13). Cronin (14) also reported a high incidence of extrusion, the risk of which is lifelong (13). In 1991 Reinsich introduced porous polyethylene implants (Medpor® Stryker, Kalmanzoo, MI, USA) for reconstructing cartilaginous facial defects, which although still in use today, are limited by high rates of infection, functional compromise such as nasal blockage and a sense of reconstructive inadequacy and dissatisfaction from the patient's perspective (13, 25–27). Particle formation followed by the subsequent inflammatory reaction also remains a concern with these implants (13).

Allografts have primarily been used for auricular and nasal reconstruction. Although avoiding the problem of donor site morbidity, early grafts suffered from significant rates of resorption (22). More recent attempts to use irradiated allogenic grafts from cadaveric donors also demonstrated over 70% resorption, as well as concerns regarding risk of disease transmission (23, 24). Concerns regarding changes in the surface topography of the graft over time and graft warping/distortion leading to deformity have also been raised (13).

## The Current “Gold Standard” for Facial Cartilaginous Reconstruction

The limitations of allografts and alloplastic implants have meant that much of the focus of modern cartilaginous auricular reconstruction has been on autologous cartilage, which was first described by Tanzer (28) and subsequently refined by Brent (29), Park (30), Nagata (31), and Firmin (15). The technique of using carved autologous costal cartilage remains the current gold standard for total auricular reconstruction worldwide today (15, 31). Smaller facial cartilaginous defects can be reconstructed using autologous cartilage grafts from either the costal, auricular or nasoseptal regions (32). The benefits of autologous reconstruction compared to synthetic implants are high biocompatibility, immunocompatibility and the ability to grow with the patient (16). However, several factors limit their utility, including the finite amount of cartilage available, associated morbidity of surgical harvest and complex three-dimensional geometry, which requires a high level of technical surgical skill (16). The risk of graft warping leading to poor long-term outcomes also remains an issue with autografts. This change in shape is thought to be due to protein polysaccharides contained within the cartilage producing tensile stresses in response to graft carving (23). It commences within the first 30 minutes after the graft is carved and can continue for weeks afterwards (23).

## Innovations in Facial Reconstructive Surgery

Auricular reconstruction is an ideal example of how refinement in surgical techniques over many years can give excellent results in expert hands. There have been various advances in the surgical approach to autologous auricular reconstruction; these include the transition towards single-stage procedures (30) as well as the use of 3D imaging and 3D models to aid surgical planning (33, 34). However, as with any autologous technique, facial cartilaginous reconstruction is limited by donor site morbidity. Engineering autologous cartilage could overcome these limitations but remains at the proof-of-concept stage and is not yet ready for widespread clinical application.

Although Buncke and Schulz (35) established the technical details surrounding microvascular ear replantation in 1964, it was not until 1980 that the first successful clinical case was reported in the literature (36). Nasal and auricular replantation using microsurgical techniques have been described in recent years with (37) and without (38, 39) venous anastomoses. Advancement of microsurgical techniques and immunosuppression made composite tissue allotransplantation a reality with the first facial transplant reported in 2005 (40).

Despite these developments, we are still confronted with shortcomings relating to the availability of donor tissues and complications of long-term immunosuppression. In order to overcome this, novel approaches have been investigated that combine advancements in nanotechnology (41), cell biology, biomaterials (42) and 3D printing (43), to engineer autologous tissues in the laboratory with a real potential for a paradigm shift in reconstructive surgery (17). Implementation of engineered autologous tissue into clinical practice could potentially eliminate the need for donor sites and their morbidity, and in addition to this reduce hospital stay and associated costs in the long term (44, 45). The surgical community worldwide is becoming increasingly aware of the importance of this field of research, and The American Society of Plastic surgeons have highlighted the need to continue to translate bench research in tissue engineering into clinical practise (46). Furthermore, the United Kingdom Government highlighted regenerative medicine as one of the “eight great technologies” worthy of significant investment with great potential to impact the health service (47).

## Native Facial Cartilage Morphology and Maturation

In order to create durable cartilage, it is important to first understand its native macro, micro, and nano-architecture (48) as well as developmental and maturation processes (16). As it is avascular, aneural, and immune-privileged, cartilage is perceived to be a relatively simple tissue to replicate (49). Cartilage consists of isolated chondrocytes within lacunae amidst extracellular matrix containing type II collagen, proteoglycans, elastic fibres, and other proteins that satisfy its structural and functional role. Articular cartilage is recognised to have a well-defined zonal organisation, where extracellular matrix and cellular organisation varies with tissue depth, and this has been shown to affect its physical properties (50). Nasoseptal cartilage has generally been assumed to be isotropic, but we know that malrotation of surgical cartilage grafts have been reported to lead to increased graft absorption or distortion (51, 52). This suggests that orientation affects the strength of the graft and indicates anisotropy which is confirmed by studies demonstrating zonal organisation, decreased cell to matrix ratio and water content but an increase in glycosaminoglycans in mature compared to immature cartilage, correlating with greater compressive stiffness (53–55).

Much like immature native cartilage, tissue engineered neocartilage, often consisting of cellular, isotropic and homogenous tissue, has also experienced problematic reabsorption rates in the literature (56, 57). The reasons for this are likely to be multifactorial, but the importance of anisotropy for engineering durable tissue cannot be overlooked, as we know it affects biomechanical strength of tissues (51, 52).

## Facial Cartilage Tissue Engineering Attempts

The landmark study of auricular tissue engineering by the Vacanti group led to the iconic image of the tissue engineered ear-shaped appendage xenografted onto the back of immunocompromised mice (56). The implanted constructs were made from bovine chondrocytes seeded onto polyglycolic acid-polylactic acid scaffolds and supported by externally fixed stents. However, once the stents were removed, the 3D shape eventually deformed, highlighting the often-overlooked features of tissue-engineered constructs, including the lack of long-term biochemical and biomechanical stability (16). This has largely been attributed to a failure in adequately maturing the constructs prior to implantation. This leads to a failure in achieving the matured anisotropic microarchitecture and thus biomechanical strength of native cartilage with its ability to withstand the forces of soft tissue cover. This paper marked the start of attempts to engineer cartilage tissue for facial reconstruction, with most studies being *in vitro* or using nude mice (58–60), with very few using immunocompetent animal models or *in vitro* maturation prior to implantation (61–63).

The biomimetic approach contrasts with these recent attempts to tissue engineer cartilage which have been based on synthetic scaffolds and unrelated mesenchymal stem cells (MSCs). This is largely because the goal by various groups involved has been to produce constructs with mechanical properties that match autologous cartilage for immediate implantation rather than supporting the cell itself to secrete autologous cartilage matrix prior to implantation. A wide variety of synthetic polymers have previously been trialled but were shown to be suboptimal due to increased susceptibility to infection, extrusion, and immune response to degradation products, all of which are reminiscent of the historical lessons of alloplastic implants (14, 64, 65). The use of synthetic nanocomposites seeded with autologous bone marrow cells for tracheobronchial transplantation have led to well-publicised controversies in the tissue engineering field (66). There have been attempts to improve biocompatibility of synthetic scaffolds. Oseni et al. for example have used nanocomposite polymer polyhedral oligomeric silsesquioxane nanocages to improve the biocompatibility and biostability of polycarbonate urethane with MSCs (67).

## First-in-man Tissue Engineered Facial Cartilage

It was more than 20 years after the Vacanti mouse when Cao's group in Beijing used implanted tissue engineered ear-shaped cartilage in a single-stage for total auricular reconstruction in five microtia patients (68). The group used chondrocytes isolated from microtia cartilage which was seeded on polycaprolactone mesh, wrapped with polyglycolic acid, and subsequently coated with polylactic acid to engineer the cartilage *in vitro* prior to its implantation. Follow up ranged from 2 months to two and a half years and revealed that four out of five cases demonstrated cartilage on post implantation biopsy. The detailed 3D structure was, however, compromised and this may be related to degradation of the inner polycaprolactone core. Longer term follow-up is required in order to establish clinical outcomes after complete degradation of the polycaprolactone core. Other scaffold-free approaches to clinical translation of tissue-engineered cartilage includes a two-stage approach. This involves cells being injected subcutaneously into the lower abdomen, and the *in-vivo* regenerated cartilage being subsequently further hand-carved into an ear-shaped framework and re-implanted into the final position (69, 70). However, with these grafts having to be carved one must question whether graft warping would occur which is an issue that has been seen with autologous grafts and must be addressed with newer technologies moving forward.

Ivan Martin's group in Basel used autologous tissue engineered constructs in a first in human trial for nasal reconstruction (71). The trial consisted of using nasal septum chondrocytes seeded onto fibrous collagen scaffolds for nasal ala reconstruction following non-melanoma skin cancer excision. In total, five adult patients were involved and follow up after 1 year revealed good patient satisfaction with both the aesthetic and functional outcomes (71). However, these were small defects and in addition no post-implantation biopsy was performed to confirm that cartilage rather than scar tissue had formed. To date, there have been no reports of tissue engineering applications for total nasal reconstruction.

## Discussion and Future Avenues

There is increasing recognition that both the cell (intrinsic) and microenvironment (extrinsic) are important for tissue engineering and that synthetic scaffolds fail to provide the necessary cues for cartilage matrix secretion (72). The importance of cells is supported by the literature, where cells alone were able to regenerate cartilage *in vivo* and formed the basis of autologous chondrocyte implantation treatments for osteoarthritis (69, 73). Other groups have used either bovine auricular chondrocytes (74) or equine auricular progenitor cells (75) to demonstrate feasibility of biomimetic approaches through seeding on natural scaffolds. Ivan Martin's group, using human nasoseptal chondrocytes and natural scaffolds (hyaluronan and collagen), recognised that *in vitro* pre-culture prior to *in vivo* implantation enhances mechanical properties of cartilage grafts (76). This may explain why this approach achieved the first successful in-human tissue engineered cartilage in facial reconstruction where other groups failed (71).

It is widely believed that to be able to engineer durable native cartilage *in vitro*, it is important to not only understand native macro- (i.e., overall shape of the tissue or organ), micro- (composition of extracellular matrix, pore shape and size vs. cell shape and size) and nanoarchitecture (nanotopography and biomolecule attachments of extracellular matrix for optimal cell adhesion and proliferation) of the tissue (48, 76), but also the developmental pathway involved in creating that architecture (77). Cartilage development is largely controlled by complex FGF-TGFβ-Wnt crosstalk that occurs *in vivo* to allow proliferation of undifferentiated MSCs prior to condensation and chondrogenic differentiation (77). Part of the developmental principle approach is to use cartilage specific progenitor/stem cells, superior to unrelated MSCs for cartilage matrix secretion (78, 79), platform technologies such as 3D bioprinting, to replicate native microscopic anisotropy and macroscopic 3D anatomy and thereby functionality (16, 45), as well as natural scaffolds or bioinks that encourage both cell adhesion and chondrogenesis (80). Induction and maintenance of maturation either through simple *in vitro* pre-culture or physiological culture conditions in bioreactors will be important for providing durability of the tissue-engineered construct (16, 45). The potential success of these approaches does, however, remain to be seen in first-in-man studies and could represent the next phase of the facial reconstruction evolution.

## Author Contributions

ZJ is the lead author of the paper and along with IW founded the idea for the review. Both contributed singificantly to the preparation of the manuscript. TD, AH, and KS assisted with the literature search whilst also contributing to the writing of the manuscript. All authors have reviewed and approved the final manuscript and agreed to be accountable for all aspects of the work.

## Conflict of Interest

The authors declare that the research was conducted in the absence of any commercial or financial relationships that could be construed as a potential conflict of interest.

## Publisher's Note

All claims expressed in this article are solely those of the authors and do not necessarily represent those of their affiliated organizations, or those of the publisher, the editors and the reviewers. Any product that may be evaluated in this article, or claim that may be made by its manufacturer, is not guaranteed or endorsed by the publisher.
